# Factors Affecting Young Adults’ Decision Making to Undergo COVID-19 Vaccination: A Patient Preference Study

**DOI:** 10.3390/vaccines10020265

**Published:** 2022-02-09

**Authors:** Gleb Donin, Anna Erfányuková, Ilya Ivlev

**Affiliations:** 1 Department of Biomedical Technology, Czech Technical University in Prague, 272 01 Kladno, Czech Republic; anna.erfanyukova@fbmi.cvut.cz; 2 Center for Health Research, Kaiser Permanente Northwest, Portland, OR 97227, USA; ilya.ivlev@kpchr.org

**Keywords:** COVID-19, discrete choice experiment, patient preferences, young adults, vaccine hesitancy, vaccine

## Abstract

Young adults are a substantial driver of lagging vaccination against COVID-19 worldwide. We aimed to understand what vaccine or vaccination environment attributes may affect young adults’ vaccine inclination. We contacted a convenience sample of 1415 students to recruit a minimum of 150 individuals for a web-based discrete choice experiment. The respondents were asked to choose one of two hypothetical vaccines, defined by six attributes—vaccine efficacy, risk of mild side effects, protection duration, administration route, recommender, and travel time to the vaccination site. Individual preferences were calculated with the Markov chain Monte Carlo hierarchical Bayes estimation. A total of 445 individuals (mean age 24.4 years, 272 (61.1%) women) completed the survey between 22 March and 3 May 2021. Vaccine protection duration (28.3 (95% CI, 27.0–29.6)) and vaccine efficacy in preventing COVID-19 (27.5 (95% CI, 26.3–28.8)) were the most important, followed by the risk of vaccine side effects (17.3 (95% CI, 16.2–18.4)). Individuals reluctant or unsure about vaccination (21.1%) prioritized the potential for mild side effects higher and vaccine efficacy lower than the vaccine-inclined individuals. New vaccination programs that target young adults should emphasize the protection duration, low risk of vaccine side effects, and high efficacy.

## 1. Introduction

Coronavirus disease 2019 (COVID-19) is associated with all-cause and COVID-19-related mortality, morbidity, and increased healthcare utilization [1,2]. Although older adults are at a higher risk for these outcomes compared with younger adults [3], young persons, particularly those with multimorbidity, are also experiencing negative effects of the current pandemic [4,5,6,7,8,9]. In addition, young adults, who returned to in-person schooling after lifting the stay-at-home policy, are likely to contribute to the spread of the virus in older adults [7,10,11].

While many individuals from high-developed countries have already been vaccinated against COVID-19, unvaccinated persons, particularly young adults, are reluctant to take up the vaccine [12,13]. Because there is no mandatory vaccination for COVID-19 for the general population, the ability to control the COVID-19 pandemic relies heavily on the success of vaccination programs [14]. The success of rapid immunization, among other factors (e.g., vaccine availability, access, infrastructure), relies on individuals’ vaccine acceptance [15]. However, high worldwide vaccine hesitancy [16,17] is forecasted to be one of the main impediments to COVID-19 vaccine uptake [18] and perhaps subsequent re-vaccination. Governments, policymakers, and health systems are facing the task of developing effective vaccine-acceptance messaging to engage individuals in vaccination [19,20], so the population can gain herd immunity [15,21]. Research on how to promote vaccination for COVID-19 is still underdeveloped [15], particularly among young adults.

Relatively high COVID-19 vaccine hesitancy [22] is observed in the general population across various countries (e.g., the US [12,23,24,25], France [18,26], and the UK and Ireland [27,28]) and specific populations (e.g., Black and Hispanic individuals, persons with no college degree, younger age, women, low income, and persons with a history of avoiding the influenza vaccine) [12,16,29,30]. A systematic review [31] of COVID-19 vaccine receptivity studies from 31 countries has shown that vaccine acceptability declined from >70% to <50% between March and October 2020 in older and younger adults. A study of vaccine hesitancy and acceptance among American medical students toward a COVID-19 vaccine found that nearly a quarter of surveyed students are reluctant to undergo vaccination immediately upon the US Food and Drug Administration’s approval [25]. A Polish survey of 1284 students found that 40.6% (521) are unwilling to be vaccinated or undecided [32]. Similar results have been shown among Italian [26], Spanish [33], German [34], and Turkish [35] students. Among the main drivers for vaccine hesitancy in students are often-cited concerns regarding adverse reactions, mistrust in the vaccine information presented by public health authorities [25,32], and overall insufficient information about the vaccine [25].

Quantitative patient preference research has been used in Europe to inform marketing authorization, reimbursement, and pricing decisions [36]. Previous vaccine preference studies were either conducted when no authorized vaccines were available [30,37] or have not focused on preferences playing an essential role in young adults [38,39]. The purpose of this study was to understand what vaccine attributes might affect young adults’ decision to undergo vaccination and explore how this information can support governments, policymakers, and health systems in developing initiatives to improve vaccination against COVID-19. We also explored factors that might serve as predictors of individuals being reluctant to undergo vaccination against COVID-19.

## 2. Materials and Methods

The protocol of this study was reviewed and approved by the Institutional Review Board (IRB ID: B1/2021) at the Faculty of Biomedical Engineering at Czech Technical University in Prague (Prague, Czech Republic).

### 2.1. Study Design

The experiment was a cross-sectional, self-administered, web-based survey conducted with a convenience sample of students aged ≥18 years from a large university in the Czech Republic between 22 March 2021 and 3 May 2021. We used Conjont.ly software (Analytics Simplified, Sydney, Australia) to elicit participants’ preferences regarding vaccination against COVID-19. We followed the International Society for Pharmacoeconomics and Outcomes Research Good Research Practices for Conjoint Analysis Task Force’s guidance [40] to develop a discrete choice experiment (DCE). We used multivariable logistic regression to identify factors that might predict young adults’ reluctance to undergo vaccination.

### 2.2. Discrete Choice Experiment Design

#### 2.2.1. Attributes and Levels

We reviewed peer-reviewed literature of previously conducted DCEs for any vaccines or earlier COVID-19 patient-preference vaccine studies to identify candidate attributes. The core set of attributes was retrieved from the two early DCE studies—conducted in the US and China—that analyzed factors associated with adults’ willingness to undergo vaccination against COVID-19 [30,41]. These studies were based on information about hypothetical vaccines. To develop levels for each attribute, we reviewed results of the first vaccine trials that had been published at the time of research design [42,43,44], websites of COVID-19 vaccine manufacturers (i.e., AstraZeneca PLC, Cambridge, UK, Moderna Inc., Cambridge, MA, USA, Pfizer Inc., New York, NY, USA) and their press releases [45,46,47], fact sheets published by the US Food and Drug Administration [48,49,50], and the World Health Organization report on vaccine development [51]. Overall, six attributes and their levels were included in the DCE (Table 1).

The “Vaccine efficacy in preventing COVID-19” attribute indicates the reduction in disease incidence in a vaccinated group compared with an unvaccinated group. The vaccine’s potential harms were grouped in the “Risk of mild side effects” attribute, which refers to the probability of short-term flu-like symptoms, for example, fatigue, headache, chills, and nausea after the vaccination. The “Protection duration” attribute reflects the time between vaccination and anticipated re-vaccination—when the vaccine provides protection against COVID-19. The “Vaccine administration route” attribute reflects the path by which a vaccine can be administered to a patient, either oral or in injections. The “Recommender of the vaccine” attribute reflects who recommended a vaccine to a respondent (i.e., family members or friends, practitioner or experts, or professional societies). The last attribute, “Time to the vaccination site,” presented an expected patient travel time to a vaccination site.

#### 2.2.2. Survey Development

**Figure 1:**
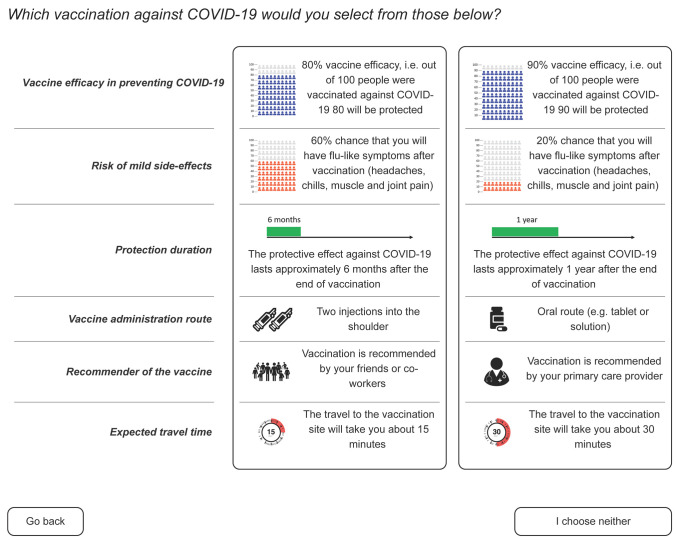
An example choice card used in the discrete choice experiment survey. Participants could magnify the pictograms.

We used the International Patient Decision Aid Standards (IPDAS) Collaboration’s guidance [52,53] on presenting numerical data to patients and ensuring an appropriate literacy level to develop a visualization of DCE choices. We consulted additional resources on the best approaches to present risk estimates to patients [54]. Levels for each attribute were presented as text or pictograms accompanied by an explanation (Figure 1). The draft survey was piloted with five volunteers. As a result of this pilot testing, we updated instructions, reordered visual elements on a webpage, and redesigned some graphics.

### 2.3. Setting, Participants, and Recruitment

The sample size for DCE studies generally ranges between 100 and 1000 participants [55,56]. We identified a minimum sample size of 150 respondents based on a number of attributes and their levels [57].

Potential participants were 1415 students enrolled in bachelor’s and master’s programs during the 2020–2021 academic year at the Faculty of Biomedical Engineering at the Czech Technical University in Prague. All students were invited via university email on 22 March 2021. The invitation email contained a description of the research, the expected time to complete a survey, information about anonymization and confidentiality, and a link to the DCE questionnaire. A reminder was sent three weeks after the initial invitation. Overall, participants had six weeks to complete the experiment.

Participants were required to indicate that they were at least 18 years old and provide informed consent to enter the study. We had no other eligibility criteria. Participation was voluntary, and no incentives were offered.

### 2.4. Experimental Design and Variables

Before participants could start the DCE, they were presented with instructions on how to complete the experiment. They were also presented with an example of a DCE question design. In the experiment, individuals were asked, “Which vaccination against COVID-19 would you select from those below?” In each choice task, respondents were asked to compare two hypothetical vaccine/vaccination scenarios (A and B) and choose their preferred one or select an “I choose nothing” option. We used Conjoint.ly to create hypothetical vaccination profiles. This algorithm tends to produce D-efficient designs rather than maximize D-efficiency [57]. The order of questions and alternatives, and the placement of respondents into choice blocks, were randomized to avoid order bias. There were 56 blocks, and each survey respondent completed one of them.

Participants who completed the DCE were also asked to provide their age, gender, ethnicity, current or anticipated pregnancy, level of education, work experience in healthcare, employment status, household income and the size of their household, the count of risk factors for severe COVID-19 (e.g., chronic kidney disease, obesity, human immunodeficiency virus) [58], previous rejection of any recommended vaccination, current COVID-19 vaccination status, prior SARS-CoV-2 infection, and experience with any vaccine’s adverse events. We used a five-point Likert scale (Strongly agree, Agree, Unsure, Disagree, and Strongly disagree) to identify participants’ willingness to undergo vaccination for COVID-19 among those who did not undergo vaccination. All questions were optional and always included the option “Prefer not to say”.

### 2.5. Analysis

#### 2.5.1. Discrete Choice Experiment

We processed the DCE choices of the respondents within the Conjoint.ly software that utilizes a Markov chain Monte Carlo hierarchical Bayes estimation to calculate individual-level preference coefficients. As the result of this analysis, relative importance scores and their 95% confidence intervals (CIs) were estimated for selected attributes, which measure how much each characteristic affects a participant’s decision to undergo vaccination. The relative importance scores were calculated as the average of each respondent attribute utility. We used McFadden’s pseudo R^2^ to assess how well our survey results describe the respondents’ answers [59]. We calculated level preference scores to understand which specific vaccination parameters were strongly preferred by the respondents.

We used participants’ self-reported sociodemographic data and stated willingness to undergo vaccination against COVID-19 to estimate attribute importance scores among subgroups. Our subgroup analyses were post hoc based on the number of respondents with a given characteristic. The subgroups were: age, gender, willingness to undergo vaccination, persons with at least one self-reported risk factor for severe COVID-19, and individuals with any experience with COVID-19 since the beginning of the pandemic (e.g., a personal history of COVID-19, a family member diagnosed with COVID-19).

#### 2.5.2. Predictors of Vaccine Hesitancy

We explored factors that might predict participants’ reluctance to undergo vaccination against COVID-19. Participants who indicated that they strongly disagreed, disagreed, or were unsure about undergoing vaccination against COVID-19 when a vaccine is available were grouped as “reluctant to be vaccinated”.

We performed a multivariable logistic regression in R (v.4.0.5) software to test the potential predictors. We used age, education, presence of risk factors for severe COVID-19, ethnicity, previous COVID-19 infection, rejection of vaccination in the past, previous vaccine adverse events, and socioeconomic index as independent variables in the regression analysis. The socioeconomic index was a ratio of reported household income (numerator) and household size (denominator). The referent and comparison groups can be found in the results section. We used the chi-square test to understand whether the variables (participants’ characteristics) were distributed differently between respondents and non-respondents. We used complete case analysis because missing data of covariate did not depend on the outcome (Supplementary Table S1) and the small size of our sample [60,61].

## 3. Results

### 3.1. Study Population

**Figure 2:**
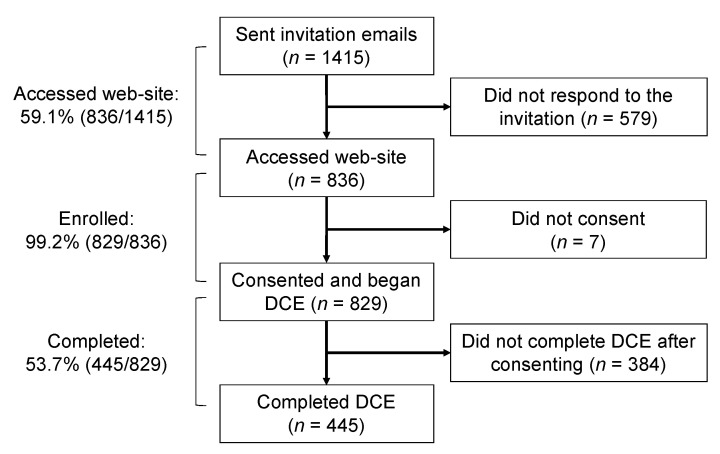
Flow diagram. DCE—discrete choice experiment.

A total of 1415 individuals were invited to participate in the study. Of these, 836 (59.1%) opened the survey link, and 829 (99.2% of those who accessed the website) enrolled (i.e., completed the consent form) (Figure 2). Fifty-four percent (445/829) of those who were enrolled completed the DCE. Of the 445 who completed the DCE, 272 (61.1%) were women, with an estimated weighted mean age of 24.4 years; 360 (80.6%) were Czech, with or without multiple ethnic backgrounds; and 264 (59.3%) had a biomedical background. A total of 77 (17.3%) individuals reported one or more risk factors for severe COVID-19, 22 (4.9%) had rejected a recommended vaccination in the past, 79 (17.6%) reported one or more adverse events with previous vaccinations, and 96 (16.4%) reported a personal history of COVID-19 infection. Only one-fifth (86, 19.3%) of participants reported being fully or partially vaccinated against COVID-19 (Table 2). The study population was representative of the general Czech young adults and university student population with respect to age, gender, and education background (see Supplementary Table S2).

Among the respondents who had not received nor started vaccination, more than two-thirds (233, 70.4%) agreed with the statement, “I want to be vaccinated against COVID-19 as soon as the vaccine is available for me.” At the same time, more than a quarter of participants (94, 28.4%) strongly disagreed, disagreed, or were unsure about getting the vaccine (Table 2).

### 3.2. Discrete Choice Experiment

**Figure 3:**
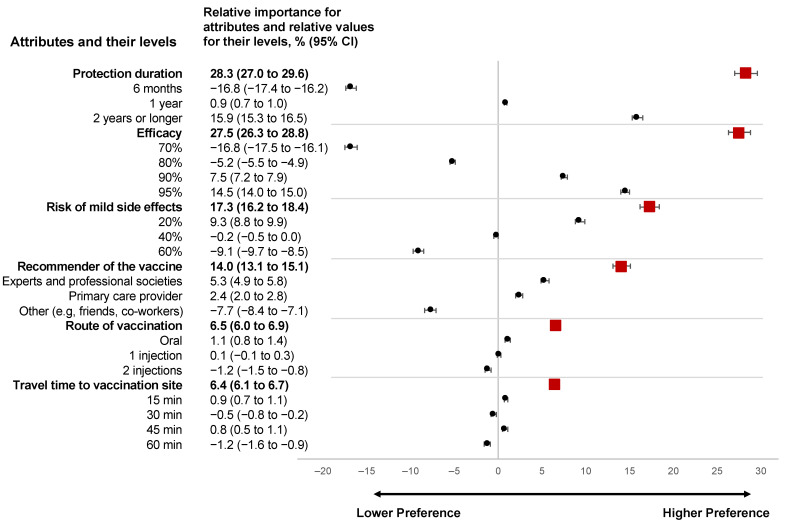
The relative importance of attributes and relative values of attribute levels. Attributes are ordered from the most important to the least important. Squares correspond to the relative importance of attributes and the lines represent 95% confidence interval. Circles correspond to the relative values of attribute levels and the lines represent 95% confidence interval.

Figure 3 demonstrates the relative attribute importance scores, relative values of attribute levels, and their 95% confidence intervals. The most important attributes for the participants were vaccine protection duration (28.3 (95% CI, from 27.0 to 29.6)) and vaccine efficacy (27.5 (95% CI, from 26.3 to 28.8)). The increase in the protection duration from six months to two years was associated with higher importance: from −16.8 (95% CI, from −17.4 to −16.2) to 15.9 (95% CI, from 15.3 to 16.5), respectively. A vaccine with a higher efficacy was more preferable—the relative value increased from a negative preference of −16.8 (95% CI, from −17.5 to −16.1) for the vaccine with 70% efficacy to a positive preference of 7.5 (95%, CI, from 7.2 to 7.90) for the vaccine with 90% efficacy. The priority for vaccines with 95% efficacy has nearly doubled (14.5 (95% CI, from 14.0 to 15.0)) compared with vaccines with 90% efficacy.

The third most important attribute was the risk of vaccine mild side effects, with a relative importance of 17.3 (95% CI, from 16.2 to 18.4). A risk of mild side effects of 40% or higher was associated with lower vaccine preference. A vaccine with a 20% risk of mild side effects was shown to be the most preferable 9.3 (95% CI, from 8.8 to 9.9).

It was also important to the participants who recommended the vaccine (14.0 (95% CI, from 13.1 to 15.1)). Experts and representatives of professional societies (5.3 (95% CI, from 4.9 to 5.8)) and a primary care provider (2.4 (95% CI, from 2.0 to 2.8)) recommending vaccination were the most important source of advice and were associated with a higher likelihood of vaccine acceptance. Advice from a family member, coworkers, and friends demonstrated the lowest impact on the decision making (−7.7 (95% CI, from −8.4 to −7.1)).

The route of vaccine administration (i.e., oral or injection) and travel time to the vaccination site were the least important attributes, 6.5 (95% CI, from 6.0 to 6.9) and 6.4 (95% CI, from 6.1 to 6.7), respectively. The participants displayed a more favorable attitude toward the oral form of vaccine (1.1 (95% CI, from 0.8 to 1.4)), compared with one injection (0.1 (95% CI, from −0.1 to 0.3)). Two injections were associated with negative preferences (−1.2 (95% CI, from −1.5 to −0.8)). There was no consistency in preferences within the increase in the expected travel time to the vaccination time, suggesting that this attribute might not hold substantial importance for young adults.

The respondents had clear preferences for vaccination attributes (McFadden’s pseudo-R^2^ = 74.7%), meaning high goodness of fit for our model.

### 3.3. DCE Subgroup Analysis

Overall, we found that persons who were reluctant or unsure about undergoing vaccination prioritized risk for mild side effects substantially higher than other participants and also displayed lower priority for vaccine efficacy (Supplementary Table S3).

We observed that vaccine efficacy was substantially less important for individuals who were reluctant to undergo vaccination against COVID-19 (23.5 (95% CI, from 21.3 to 25.8); *n* = 94) than those inclined toward vaccination (28.7 (95% CI, from 26.9 to 30.5); *n* = 233), without risk factors for severe COVID-19 (27.9 (95% CI, from 26.4 to 29.3); *n* = 334), and with a biomedical background (28.2 (95% CI, from 26.6 to 30.1); *n* = 264).

Individuals reluctant or unsure about undergoing vaccination prioritized the risk for mild side effects (21.8 (95% CI, from 19.5 to 24.5)) substantially higher than individuals from other subgroups. For example, the relative attribute importance score was 16.3 (95% CI, from 14.9 to 17.7) for those who were willing to undergo vaccination (*n* = 233), 16.9 (95% CI, from 15.7 to 18.2) for those without risk factors for severe disease (*n* = 334), 16.9 (95% CI, from 15.7 to 18.2) for those with a personal history of COVID-19 (*n* = 96), and those with a biomedical background (17.3 (95% CI, from 15.8 to 18.8); *n* = 264).

### 3.4. Predictors of Vaccine Hesitancy

Overall, 238 persons contributed data for the analysis. Persons with missing responses (*n* = 89) were similar to those with complete responses for all characteristics, except for age. There was a smaller proportion of persons aged 18–24 years among those with complete data (Supplementary Table S1).

Individuals who reported rejecting any recommended vaccine in the past, before the COVID-19 pandemic, had a 3.30-fold (odds ratio, OR) increased risk (95% CI, from 1.06 to 10.31) of being reluctant to undergo vaccination against COVID-19 (Table 3). We have not observed differences among persons from different age groups, those reporting different gender, biomedical background, presence of risk factors for severe disease, a personal history of vaccine side effects, those with different socioeconomic indexes, or levels of education.

## 4. Discussion

Young adults are less likely to accept the COVID-19 vaccine than their older counterparts in many countries [16,17]. The vaccination rate against COVID-19 in young adults is already behind that in older adults [62]. New and innovative vaccination programs need to be developed to accelerate vaccination among vaccine-hesitant individuals. Our study provides insight into the vaccine and vaccination attributes important to young adults.

Our study demonstrated that a vaccine’s protective duration and efficacy are the most important parameters for young adults. Young adults who displayed a hesitancy to undergo vaccination against COVID-19 emphasized the risk of mild side effects and less on vaccine efficacy than those inclined to uptake vaccination. Even though some common factors affect younger and older adults’ decision making when deciding to undergo vaccination (i.e., vaccine efficacy, the risk of side effects), other factors and their importance differ from older adults.

A few large population-based population preferences studies have been conducted in the US and the UK (*n* = 6457) [30,38,63]. These studies tested various attributes of vaccines and vaccination programs to understand what attributes might play an important role in patients’ decision making. Similar to our study, in two US- and UK-based studies, vaccine efficacy was shown to be the most critical attribute, and higher efficacy was associated with a higher probability of vaccination [30,38]. A large US-based study with 1971 adults also concluded that a longer vaccine protection time is seen as more preferable, and a lower risk for side effects increases the probability of vaccination [30]. Our study suggests that anticipated travel time to the vaccination site might not play an important role among young adults. None of the other known to us studies tested the importance of the anticipated travel time to the vaccination site. A less intensive vaccination (i.e., one injection vs. two, oral form of vaccination vs. injections) was preferable among our participants. Similarly, American adults demonstrated a substantial negative preference for an annual vaccine booster (i.e., increased vaccination intensity) rather than a one-time vaccination and for two vaccine doses rather than one dose [30,63]. Young adults displayed a similar trend, preferring oral vaccine administration rather than one or two injections. These studies also add a better understanding of the importance of other attributes not tested in our experiment. For example, one US-based study demonstrated that mandatory vaccination was less preferable than voluntary vaccination [63]. American adults are less likely to undergo vaccination if the vaccine was made outside of the US, particularly if it originated from China [30]. It has also been demonstrated that the vaccination site can be an important attribute. Thus, in two studies, participants displayed a higher likelihood of vaccine uptake if vaccination was conducted at their general provider’s office or a health facility rather than at a mass vaccination site or a mobile vaccination unit [38,63].

Our study demonstrated that personal history of ever rejecting a recommended vaccination is associated with higher odds of reluctance toward vaccination against COVID-19 (OR, 3.30 (95% CI, from 1.06 to 10.31)). This result was similar in two US studies with 3279 adults. In one study, a history of flu vaccination in the past was associated with a 4.70-fold (from 3.55 to 6.23) increased odds of willingness to undergo vaccination against COVID-19 [64]. In another study, not having an influenza vaccine in the past year was independently associated with vaccine hesitancy [23]. Personal history of previous influenza vaccination was associated with an increased likelihood of vaccination against COVID-19 (OR, 2.74 (95% CI, from 2.12 to 3.57)) in the Czech study with 3550 healthcare workers [65]. None of the other factors in our study (e.g., gender, risk factors for severe COVID, history of side effects associated with vaccination, socioeconomic status) were shown to predict vaccination hesitancy. The above-mentioned Czech study [65] has demonstrated that female gender (OR, 0.58 (95% CI, from 0.45 to 0.75)), presence of chronic health conditions (OR, 0.80 (95% CI, from 0.66 to 0.97)), and a personal history of COVID-19 (OR, 0.41 (95% CI, from 0.34 to 0.49)) were associated with lower odds of undergoing vaccination.

A broader literature has also shown that vaccination against COVID-19 was politicized, and belonging to a political party might affect individuals’ decision making for vaccination [30,63]. Conspiracy beliefs also play an important role in persons being reluctant to undergo vaccination [66].

### 4.1. Future Research

Vaccine hesitancy for COVID-19 in the Czech Republic and many other developed countries still remains understudied for children and adolescents, racial and ethnic minorities, socioeconomically disadvantaged individuals, individuals affected by a disability, those who experience homelessness, and representatives of other minority groups. More research is needed to understand individuals’ needs (e.g., information, infrastructure) to maximize the vaccination rate, particularly among underserved populations. We will target these special populations in our future population-based research.

### 4.2. Limitations

Our study has several limitations. First, we used a convenience sample of university students, which limits the generalizability of our findings to all Czech young adults. We demonstrated that the respondents were representative of the Czech young adults in terms of age, gender, and education (Supplementary Table S2). Second, not all information was known about the currently available vaccines when the DCE was designed. For example, a 20% risk of side effects and 95% vaccine effectiveness for two years could be unachievable. Third, this study was conducted early in the COVID-19 pandemic. Results from more recent studies might provide new information. Fourth, our results might differ from actual choices due to the nature of the DCE and the inability to account for real-life behavior. Finally, the subgroup analysis for vaccine preferences was ad hoc; the sample size of some subgroups was smaller than the calculated minimum for our DCE.

## 5. Conclusions

This study demonstrated that protection duration, vaccine efficacy, and the potential for mild side effects play an important role in deciding whether to undergo vaccination against COVID-19 among young adults. Expected travel time to the vaccination site might not be an important attribute. Individuals reluctant or unsure about vaccination prioritized the potential for mild side effects higher and vaccine efficacy lower than vaccine inclined individuals. Our results can be used to design vaccination strategies to increase the vaccination rate among young adults.

## Figures and Tables

**Table 1:** Attributes and levels for hypothetical COVID-19 vaccines and vaccination environment.

Attributes	Levels
Efficacy in preventing COVID-19	70%; 80%; 90%; 95%
Risk of mild side effects	20%; 40%; 60%
Protection duration	6 months; 1 year; 2 years
Vaccine administration route	Oral; 1 injection; 2 injections
Recommender of the vaccine	Experts and professional societies; primary care provider; family or friends
Expected travel time to the vaccination site	15 min; 30 min; 45 min; 60 min

**Table 2:** Participants’ characteristics.

Characteristics	All Participants, *n* (%) *n* = 445	Reluctant to Undergo Vaccination for COVID-19, *n* (%) ^a^ *n* = 94	Inclined to Undergo Vaccination for COVID-19, *n* (%) ^b^ *n* = 233
Age, years			
18–24	305 (68.5)	71 (75.5)	177 (76.0)
25–34	83 (18.7)	16 (17.0)	41 (17.6)
≥35	28 (6.3)	7 (7.4)	15 (6.4)
PNTS or no answer	29 (6.5)	0 (0.0)	0 (0.0)
Gender			
Woman	272 (61.1)	64 (68.1)	151 (64.8)
Man	142 (31.9)	30 (31.9)	79 (33.9)
PNTS or no answer	31 (7.0)	0 (0.0)	3 (1.3)
Ethnicity ^c^			
Czech or multiple, including Czech	370 (80.6)	79 (80.6)	212 (88.7)
Moravian	17 (3.7)	4 (4.1)	7 (2.9)
Slovak	19 (4.1)	4 (4.1)	11 (4.6)
Not listed	12 (2.7)	5 (5.1)	4 (1.7)
PNTS or no answer	41 (8.9)	6 (6.1)	5 (2.1)
Education background			
Primary education	1 (0.2)	0 (0.0)	1 (0.4)
Secondary education	258 (58.0)	62 (66.0)	157 (67.4)
Post-secondary education	7 (1.6)	3 (3.2)	3 (1.3)
Bachelor or equivalent	137 (30.8)	27 (28.7)	63 (27.0)
Master’s or equivalent	8 (1.8)	2 (2.1)	4 (1.7)
PhD or another doctorate	5 (1.1)	0 (0.0)	4 (1.7)
PNTS or no answer	29 (6.5)	0 (0.0)	1 (0.4)
Biomedical background			
Yes	264 (59.3)	56 (59.6)	134 (57.5)
No	139 (31.2)	35 (37.2)	88 (37.8)
PNTS or no answer	42 (9.4)	3 (3.2)	11 (4.7)
Employment status			
Student	307 (69.0)	74 (78.7)	188 (80.7)
Employee	87 (19.6)	15 (16.0)	31 (13.3)
Self-employed/Entrepreneur	7 (1.6)	2 (2.1)	5 (2.1)
Unemployed	7 (1.6)	2 (2.1)	5 (2.1)
Other	5 (1.1)	0 (0.0)	3 (1.3)
PNTS or no answer	32 (7.2)	1 (1.1)	1 (0.4)
Annual gross household income, EUR ^d^			
<6900	67 (15.1)	19 (20.2)	36 (15.5)
6900–13,900	54 (12.1)	11 (11.7)	28 (12.0)
13,900–20,800	66 (14.8)	12 (12.8)	36 (15.5)
20,800–30,100	54 (12.1)	11 (11.7)	28 (12.0)
30,100–37,000	44 (9.9)	7 (7.4)	33 (14.2)
37,000–41,700	14 (3.1)	3 (3.2)	5 (2.1)
41,700–46,300	9 (2.0)	2 (2.1)	4 (1.7)
>46,300	19 (4.3)	4 (4.3)	12 (5.2)
PNTS or no answer	118 (26.5)	25 (26.6)	51 (21.9)
Socioeconomic index (SEI) ^e^			
<33rd percentile (lowest SEI)	115 (25.8)	61 (26.2)	30 (31.9)
≥33rd percentile (highest SEI)	200 (44.9)	114 (48.9)	37 (39.4)
No answer	130 (29.2)	58 (24.9)	27 (28.7)
Pregnancy (percent of women)			
Yes	2 (0.7)	1 (1.6)	1 (0.7)
Planning within one year	9 (3.3)	5 (7.8)	2 (1.3)
No	260 (95.6)	58 (90.6)	147 (97.4)
No answer	1 (0.4)	0 (0.0)	1 (0.7)
Risk factors for severe COVID-19 ^f^			
One or more	77 (17.3)	13 (13.8)	44 (18.9)
None	334 (75.1)	78 (83.0)	187 (80.3)
PNTS or no answer	34 (7.6)	3 (3.2)	2 (0.9)
Rejected any recommended vaccine in the past			
Yes	22 (4.9)	12 (12.8)	10 (4.3)
No	387 (87.0)	79 (84.0)	221 (94.8)
PNTS or no answer	36 (8.1)	3 (3.2)	2 (0.9)
Adverse events with any vaccine (not against COVID-19) ^c^			
Allergic reaction	12 (2.7)	5 (5.2)	5 (2.1)
Other adverse events	67 (14.9)	17 (17.5)	28 (11.9)
No or not sure	339 (75.3)	73 (75.3)	201 (85.5)
PNTS or no answer	32 (7.1)	2 (2.1)	1 (0.4)
History of COVID-19 ^c^			
Personal	96 (16.4)	21 (17.5)	52 (16.6)
Relatives	172 (29.4)	34 (28.3)	102 (32.5)
Other (e.g., friends, coworkers)	203 (34.6)	38 (31.7)	113 (36.0)
No or not sure	84 (14.3)	24 (20.0)	46 (14.6)
PNTS or no answer	31 (5.3)	3 (2.5)	1 (0.3)
Vaccinated against COVID-19			
Yes, first dose or fully	86 (19.3)	Not Applicable	Not Applicable
No	331 (74.4)	94 (100)	233 (100)
PNTS or no answer	28 (6.3)	Not Applicable	Not Applicable
Agreement with the statement “I want to be vaccinated against COVID-19 as soon as the vaccine is available for me.” ^g^			
Strongly agree	140 (42.3)	Not Applicable	140 (60.1)
Agree	93 (28.1)	Not Applicable	93 (39.9)
Not sure	54 (16.3)	54 (57.4)	Not Applicable
Disagree	23 (6.9)	23 (24.5)	Not Applicable
Strongly disagree	17 (5.1)	17 (18.1)	Not Applicable

PNTS—prefer not to say. ^a^ Individuals who answered “Strongly disagree,” “Disagree,” or “Unsure” to the statement “I want to be vaccinated against COVID-19 as soon as the vaccine is available for me.” ^b^ Individuals who answered “Strongly agree” or “Agree” to the statement “I want to be vaccinated against COVID-19 as soon as the vaccine is available for me.” ^c^ Respondents could select several categories—the sum does not add to 100%. ^d^ We used the average Czech koruna to Euro exchange rate from the Czech national bank for April 2021, EUR 1 = CZK 25.924, rounded off to the nearest hundred. ^e^ A ratio of reported household income (numerator) and household size (denominator). ^f^ Any set of the following: cancer, chronic liver disease; chronic obstructive pulmonary disease; cardiovascular diseases (e.g., heart failure, ischemic heart disease, cardiomyopathy); weakened immune system (e.g., after organ transplantation, human immunodeficiency virus, etc.); obesity; sickle cell disease; smoking; type 2 diabetes mellitus. ^g^ Among those who have not started or completed vaccination against COVID-19.

**Table 3:** Results of multivariable logistic regression for sociodemographic predictors of reluctance to undergo vaccination against COVID-19 (*n* = 238).

Participants’ Characteristics	Reluctance to Undergo Vaccination	
	Adjusted Odds Ratio (95% CI)	*p*
Age, years		
18–24	0.99 (0.43–2.28)	0.975
≥25	Referent	
Gender		
Man	1.06 (0.55–2.03)	0.852
Woman	Referent	
Biomedical background		
No	1.65 (0.89–3.10)	0.114
Yes, any	Referent	
Risk factors for severe COVID-19 ^a^		
≥1	1.56 (0.72–3.65)	0.282
None	Referent	
Adverse events with any vaccine (not against COVID-19)		
Yes	1.62 (0.72–3.52)	0.233
No	Referent	
Rejected any recommended vaccination in the past (not against COVID-19)		
Yes	3.30 (1.06–10.31)	0.037
No	Referent	
Socioeconomic index (SEI)		
<33rd percentile (lowest SEI)	1.51 (0.81–2.80)	0.191
≥33rd percentile (highest SEI)	Referent	
Education		
Bachelor degree or higher	0.75 (0.35–1.56)	0.446
Post-secondary or lower	Referent	

^a^ Any set of the following: cancer, chronic liver disease; chronic obstructive pulmonary disease; cardiovascular diseases (e.g., heart failure, ischemic heart disease, cardiomyopathy); weakened immune system (e.g., after organ transplantation, human immunodeficiency virus, etc.); obesity; sickle cell disease; smoking; type 2 diabetes mellitus.

## Data Availability

The datasets used and/or analyzed during the current study are available from the corresponding author on reasonable request.

## Supplementary Materials

The following are available online at https://www.mdpi.com/article/10.3390/vaccines10020265/s1, Table S1: Comparison of respondents with and without missing data for multivariable logistic regression, Table S2: The age, gender, and educational background of the study population and of the general Czech students’ population, Table S3: Subgroup estimates of relative attribute importance scores.
