# Supplementary material for: Factors Affecting Young Adults’ Decision Making to Undergo COVID-19 Vaccination: A Patient Preference Study
Source: Vaccines (Basel). 2022 Feb 9;10(2):265. doi: 10.3390/vaccines10020265 (PMC8878672; doi:10.3390/vaccines10020265)
Supplement: Supplementary file 1 [file vaccines-10-00265-s001.zip › vaccines-1513866-supplementary.pdf]

Table S1. Comparison of respondents with and without missing data for multivariable logistic regression

Participants' characteristics	Persons with complete data, n (%) n = 238	Persons with missing data,[a] n (%) n = 89	*p*-value[b]
Age = 18–24 years	173 (72.7)	75 (84.3)	0.042
Gender = Woman	82 (34.5)	27 (31.4)	0.703
Biomedical background = No	90 (37.8)	33 (44.0)	0.412
Risk factors for severe COVID-19[c] = No	192 (80.7)	73 (86.9)	0.263
Adverse events with any vaccine (not COVID-19) = Yes	37 (15.5)	13 (15.1)	1.000
Rejected any recommended vaccine in the past = Yes	16 (6.7)	6 (7.1)	1.000
Socioeconomic index (SEI)[d] = less than 33rd percentile (lowest SEI)	87 (36.6)	4 (40.0)	1.000
Education background = Bachelor's degree or higher	83 (34.9)	23 (26.1)	0.173

The analysis was performed among those who agreed, disagreed or was unsure with the statement "I want to be vaccinated against COVID-19 as soon as the vaccine is available for me" (n=327).

[a] Data were omitted from the analysis if one or more covariates were missing for respondent.

[b] Difference between groups were examined with the chi-squared test for proportions.

[c] Any set of the following: cancer, chronic liver disease; chronic obstructive pulmonary disease; cardiovascular diseases (e.g., heart failure, ischemic heart disease, cardiomyopathy); weakened immune system (e.g., after organ transplantation, human immunodeficiency virus, etc.); obesity; sickle cell disease; smoking; type 2 diabetes mellitus.

[d] A ratio of reported household income (numerator) and household size (denominator).

Table S2. The age, gender, and education background of the study population and of the general Czech students' population

<table>
<tr><th>Characteristics</th><th>Study, n (%)
N=445</th><th>Czech students in 2020,[a]
n (%)
N=299,396</th><th>Czech young adults (18–34),[b]
n (%)
N=2,034,700</th></tr>
<tr><td>Age, years</td><td></td><td></td><td></td></tr>
<tr><td>18–24</td><td>305 (68.5)</td><td>202,315 (67.6)</td><td>676,900 (33.3)</td></tr>
<tr><td>25–34</td><td>83 (18.7)</td><td>70,611 (23.6)</td><td>1,357,800 (66.7)</td></tr>
<tr><td>35–44</td><td>13 (2.9)</td><td rowspan="3">26,300 (8.8)</td><td></td></tr>
<tr><td>45–54</td><td>12 (2.7)</td><td></td></tr>
<tr><td>55–64</td><td>3 (0.7)</td><td></td></tr>
<tr><td>Prefer not to say or no answer</td><td>29 (6.5)</td><td></td><td></td></tr>
<tr><td>Sex (for Czech students)/Gender (for our study)</td><td></td><td></td><td></td></tr>
<tr><td>Female/Woman</td><td>272 (61.1)</td><td>166,181 (55.5)</td><td>986,600 (48.5)</td></tr>
<tr><td>Male/Man</td><td>142 (31.9)</td><td>133,215 (44.5)</td><td>1,048,100 (51.5)</td></tr>
<tr><td>Prefer not to say or no answer</td><td>31 (7.0)</td><td></td><td></td></tr>
<tr><td>Education background</td><td></td><td></td><td></td></tr>
<tr><td>Primary education</td><td>1 (0.2)</td><td></td><td>309,700 (15.2)</td></tr>
<tr><td>Secondary education</td><td>258 (58.0)</td><td rowspan="2">179,594 (60.0)</td><td>1,227,800 (60.3)</td></tr>
<tr><td>Post-secondary education</td><td>7 (1.6)</td><td></td></tr>
<tr><td>Bachelor's or equivalent</td><td>137 (30.8)</td><td>69,949 (23.3)</td><td>496,400 (24.4)</td></tr>
<tr><td>Master's or equivalent</td><td>8 (1.8)</td><td></td><td></td></tr>
<tr><td>PhD or equivalent</td><td>5 (1.1)</td><td></td><td></td></tr>
<tr><td>Prefer not to say or no answer</td><td>29 (6.5)</td><td></td><td></td></tr>
</table>

[a] - according to Department of Statistics, Analysis and Development of Education, The Ministry of Education of the Czech Republic. Available at https://dsia.msmt.cz//vystupy/vu_vs_f1.html

[b] - according to EuroStat Data. Last update: 13/10/2021. Available at https://ec.europa.eu/eurostat/databrowser/view/EDAT_LFS_9901/default/table?lang=en

Table S3. Subgroup estimates of relative attribute importance scores

Subgroups	Protection duration, % (95% CI)	Efficacy, % (95% CI)	Risk of mild side effects, % (95% CI)	Recommender, % (95% CI)	Route of vaccination, % (95% CI)	Travel time to a vaccination site, % (95% CI)
Aged 18–24 years (n=305)	28.4 (26.9 to 29.9)	27.4 (26.0 to 28.8)	17.2 (15.9 to 18.5)	14.2 (13.0 to 15.5)	6.5 (6.0 to 7.1)	6.2 (5.9 to 6.6)
Aged ≥ 25 years (n=111)	28.3 (25.9 to 30.6)	28.3 (25.8 to 31.0)	17.4 (15.4 to 19.5)	13.1 (11.3 to 15.0)	6.4 (5.7 to 7.2)	6.5 (5.9 to 7.2)
Women (n=272)	28.4 (26.7 to 29.9)	27.5 (25.9 to 29.1)	17.5 (16.0 to 18.8)	14.0 (12.6 to 15.3)	6.3 (5.8 to 6.8)	6.3 (6.0 to 6.7)
Men (n=142)	28.5 (26.2 to 30.7)	27.8 (25.7 to 30.0)	16.8 (14.6 to 18.9)	13.9 (12.3 to 15.7)	6.8 (6.1 to 7.5)	6.3 (5.7 to 6.9)
With any biomedical background (n=264)	28.5 (27.0 to 30.1)	28.2 (26.6 to 30.1)	17.3 (15.8 to 18.8)	13.1 (11.9 to 14.4)	6.5 (6.0 to 7.0)	6.3 (6.0 to 6.7)
Without biomedical background (n=139)	27.8 (25.6 to 30.0)	26.6 (24.8 to 28.8)	17.4 (15.5 to 19.3)	15.4 (13.6 to 17.1)	6.5 (5.7 to 7.3)	6.4 (5.9 to 7.0)
With ≥1 risk factors for severe COVID-19 (n=77)	26.4 (23.6 to 29.3)	26.8 (23.9 to 29.5)	18.5 (16.0 to 21.0)	14.7 (12.3 to 17.2)	6.7 (5.8 to 7.7)	6.9 (6.1 to 7.7)
Without risk factors for severe COVID-19 (n=334)	28.9 (27.4 to 30.4)	27.9 (26.4 to 29.3)	16.9 (15.7 to 18.2)	13.7 (12.6 to 14.9)	6.4 (6.0 to 6.9)	6.2 (5.9 to 6.5)
Reluctant or unsure about undergoing vaccination against COVID-19 (n=94)	26.9 (24.0 to 29.7)	23.5 (21.3 to 25.8)	21.8* (19.5 to 24.5)	14.4 (12.4 to 16.5)	6.9 (6.0 to 7.9)	6.4 (5.7 to 7.0)
Who are willing to undergo vaccination against COVID-19 (n=233)	28.9 (27.1 to 30.6)	28.7 (26.9 to 30.5)	16.3 (14.9 to 17.7)	13.6 (12.4 to 14.9)	6.5 (6.0 to 7.1)	6.0 (5.6 to 6.4)
Any COVID-19 experience (n=330)	27.4 (24.7 to 30.0)	26.9 (24.1 to 29.7)	18.6 (16.3 to 21.0)	14.3 (11.9 to 16.8)	6.5 (5.7 to 7.5)	6.3 (5.6 to 6.9)

With personal history of COVID-19 (n=96)	28.7 (27.1 to 30.3)	27.8 (26.4 29.2)	16.9 (15.7 to 18.2)	13.9 (12.8 to 15.1)	6.4 (5.9 to 7.0)	6.3 (6.0 to 6.6)
Without personal history of COVID-19 (n=84)	29.2 (26.5 to 32.0)	26.8 (24.2 to 29.1)	17.4 (15.1 to 19.7)	14.3 (12.2 to 16.6)	6.2 (5.5 to 7.0)	6.3 (5.7 to 6.9)
